# In vitro antioxidant activities and inhibitory effects of phenolic extract of *Senecio biafrae* (Oliv and Hiern) against key enzymes linked with type II diabetes mellitus and Alzheimer's disease

**DOI:** 10.1002/fsn3.749

**Published:** 2018-08-13

**Authors:** Basiru O. Ajiboye, Oluwafemi A. Ojo, Marry A. Okesola, Ayodele J. Akinyemi, Justina Y. Talabi, Olajumoke T. Idowu, Adewale O. Fadaka, Aline A. Boligon, Marli M. Anraku de Campos

**Affiliations:** ^1^ Department of Chemical Sciences Afe Babalola University Ado‐Ekiti Nigeria; ^2^ Department of Human Nutrition and Dietetics Afe Babalola University Ado‐Ekiti Nigeria; ^3^ Graduate Program in Pharmaceutical Sciences Federal University of Santa Maria Santa Maria Brazil

**Keywords:** α‐amylase, α‐glucosidase, acetylcholinesterase, butrylcholinesterase, *Senecio biafrae* leaves

## Abstract

The phenolic extract of *Senecio biafrae* leaves was investigated to determine the in vitro antioxidant, phenolic profiles, and inhibition of key enzymes relevant to type II diabetes mellitus (α‐amylase and α‐glucosidase) and Alzheimer's disease (acetylcholinesterase and butrylcholinesterase). The phenolic extract demonstrated significant scavenging abilities against all in vitro antioxidant parameters assessed. Reversed‐phase HPLC of the extract revealed the presence of gallic acid, chlorogenic, caffeic acid, rutin, quercetin, and kaempferol. The extract also inhibited activities of α‐amylase (IC_50_ = 126.90 μg/ml), α‐glucosidase (IC_50_ = 139.66 μg/ml), acetylcholinesterase (IC_50_ = 347.22 μg/ml), and butrylcholinesterase (IC_50_ = 378.79 μg/ml), which may be attributed to the antioxidant potential of the extract and its phenolic composition. Therefore, this study suggests that the leaves of *S. biafrae* may be useful in the management of diabetes mellitus and Alzheimer's disease.

## 1 INTRODUCTION

Diabetes mellitus (DM) is a global disease affecting millions of people and is ranked seventh leading cause of death (Danaei et al., 2011). DM has been associated with hyperglycemia, which is the primary cause of most diabetes complications (Shodehinde & Oboh, 2012). Oritz et al. (2007) reported that hyperglycemia is a condition of abnormal increase in plasma glucose level, and in type II diabetes mellitus is due to insulin resistance which may be due to a number of defects in signal transduction ranging from abnormal insulin or insulin receptors to defects in glucose transporters. Persistent hyperglycemia can lead to increase generation of reactive oxygen species (ROS) with a decrease in endogenous antioxidants (Oritz et al., 2007). An effective approach for type II diabetes mellitus management has been through inhibitions of pancreatic α‐amylase and intestinal α‐glucosidase enzymes as documented by Krentz and Bailey (2005). Kwon, Vattem, and Shetty (2006) reported that intestinal α‐glucosidase inhibition, delays the absorption of glucose, moderates postprandial blood glucose elevation, and thus mimics the effects of dieting on hyperglycemia. Also, amylase inhibition may be useful in the management of type II diabetes mellitus and obesity (Custódio et al., 2015). However, acarbose, miglitol, and voglibose which are drugs currently used as α‐amylase and α‐glucosidase inhibitors demonstrate side effects such as abdominal distension, bloating, meteorism, and flatulence (Chakrabarti & Rajagopalan, 2002). Therefore, natural inhibitors from dietary plants inhibited both α‐amylase and α ‐glucosidase and can be used as remedy for postprandial hyperglycemia with no or minimal side effects (Kwon et al., 2006; Shodehinde & Oboh, 2012).

Alzheimer's disease (AD) accounts for 60%–80% of all cases of dementia, and it has no cure and is the fourth leading cause of death in developed nations, after heart disease, cancer, and stroke (Custódio et al., 2015). According to Alzheimer's Disease International (2009), more than 36 million people in 2010 are suffering from AD, and it has been projected that more than 66 million people will be suffering from AD by 2030, if necessary actions are not taken. AD has been describe by a reduction in the levels of the neurotransmitteracetylcholine (ACh), which is hydrolyzed mainly by acetylcholinesterase (AChE) and then by butrylcholinesterase (BuChE) (Racchi, Mazzucchelli, Porrello, Lanni, & Govoni, 2004). Moreover, the inhibition of AChE is currently the most established approach for the management of AD, and numerous AChE inhibitors (AChEI) are used to attenuate the symptoms associated with this disease (such as tacrine, donzepil, and galanthamine) as reported by Orhan, Kartal, Tosun, and Sener (2007). However, these AChEI are characterize with hepatotoxicity, dizziness, vomiting, and diarrhea among others, and this drives the needs to search for novel and safer therapy (Custódio et al., 2015).

Alam et al. (2014) reported that AD is related to type II diabetes mellitus (T2D) and that both diseases share the same pathophysiology, leading to the hypothesis that AD might be type III diabetes. This is based on emerging similarities between the two diseases, which include protein conformational disorders; association with obesity, insulin resistance, inflammation, and endoplasmic reticulum stress; en‐route initiation; and/or stage aggravation (Priyadarshini, Kamal, & Nigel, 2012). Patients with hyperinsulinemia, insulin resistance and T2D are at an increased risk of memory impairment and AD (Kroner, 2009). Thus, Nagarani et al. (2009) reported that antidiabetic drugs might be beneficial in treating patients with AD.

*Senecio biafrae* (Oliv and Hiern), called “worowo” in the Western part of Nigeria, is a vegetable; grown mostly under cocoa tree plantation. This plant leaf is believed to be endowed with medicinal properties (Ajiboye et al., 2014). Ajiboye, Ibukun, Edobor, Ojo, and Onikanni (2013) documented the phytochemical constituents of the plant's leaf, with high content of phenolic compounds. The uses of phenolic extract of *S. biafrae* leaf in vitro in the management of type II diabetes mellitus and Alzhimer's disease are scanty in the literature. Therefore, this study was designed to investigate the antioxidant effect and possible inhibition against key enzymes linked to type II diabetes mellitus (α‐amylase and α‐glucosidase) and Alzheimer's disease (acetylcholinesterase and butrylcholinesterase) of the plant phenolic extract.

## 2 MATERIALS AND METHODS

### 2.1 Plant material

Fresh sample of *S. biafrae* leaf was purchased from Oja Oba Market in Ado‐Ekiti, Ekiti State, Nigeria. The plant was identified and authenticated at the herbarium unit of the Plant Biology, Ekiti State University, Nigeria with voucher specimen number UHAE 138. The plant sample was washed with distilled water and shade‐dried for 2 weeks at 25°C. Thereafter, it was ground to a fine powder, and stored in the refrigerator before phenolic extraction.

### 2.2 Preparation of phenolic extract

Fifty grams of the powdered sample was extracted with 80% acetone (1:5 w/v) and filtered. The filtrate was evaporated using a rotary evaporator under vacuum at 45°C until 90% of the filtrate had been dried and subsequently lyophilized to obtain dry weight extract. The extract was kept in a freezer prior to further analysis (Chu, Sun, Wu, & Liu, 2002).

### 2.3 Determination of total phenolic content

Appropriate dilutions of the extracts were oxidized with 2.5 ml of 10% Folin‐Ciocalteau's reagent (v/v) and neutralized by 2.0 ml of 7.5% sodium carbonate. The reaction mixture was incubated for 40 min at 45°C and the absorbance was read at 765 nm (Singleton, Orthofer, & Lamuela, 1999). The total phenol content was calculated using gallic acid as the standard.

### 2.4 Determination of total flavonoid content

Briefly, 0.5 ml of appropriately diluted extract was mixed with 0.5 ml of methanol, 50 μl of 10% AlCl_3_, 50 μl of 1 M potassium acetate and 1.4 ml of distilled water. This was incubated at room temperature for 30 min and subsequently measured at 415 nm. The total flavonoid was calculated using quercetin as standard (Meda, Lamien, Romito, Millogo, & Nacoulma, 2005).

### 2.5 HPLC‐DAD analyses

*Senecio biafrae* extracts at 10 mg/ml were injected onto reversed‐phase Phenomenex C_18_ column (4.6 mm × 250 mm) packed with 5 μm diameter particles. The mobile phases were 0.5% (v/v) aqueous formic acid (solvent A) and 1% (v/v) acetic acid in acetonitrile (solvent B). The binary elution system was as follows: 2% B for 5 min to wash the column, a linear gradient was 8% B (25 min), 12% B (45 min), 24% B (60 min). After 80 min, the organic phase concentration was reduced to 2% (B) and lasted 10 min for column equilibration. Flow rate of 0.6 ml/min and injection volume of 40 μl (Carvalho et al., 2016) were used. Quantifications were carried out by integration of the peaks using the external standard method, at 254 nm for gallic acid, 327 nm for chlorogenic and caffeic acid, and 366 nm for rutin, quercetin and kaempferol. The extract and mobile phase was filtered through 0.45 μm membrane filter (Millipore) and then degassed by ultrasonic bath prior to use. Stock solutions of standards references were prepared in the HPLC mobile phase at a concentration range of 0.025–0.300 mg/ml. Chromatography peaks were confirmed by comparing retention times with those of reference standards and by DAD spectra (200–700 nm). All chromatography operations were carried out at ambient temperature in triplicate.

### 2.6 2,2‐diphenyl‐1‐picrylhydrazyl (DPPH) free radical scavenging ability

Briefly, 1 ml of the extract was mixed with 1 ml of 0.4 mM methanolic solution containing DPPH radicals. Then, the mixture was left in a dark for 30 min and the absorbance was measured at 516 nm (Gyamfi, Yonamine, & Aniya, 1999).

### 2.7 2,2'‐azobis‐3‐ethylbenzothiazoline‐6‐sulfonate (ABTS) radical scavenging ability

The method of Re et al. (2009) was employed. ABTS was generated by reacting 7 mM of ABTS aqueous solution with K_2_S_2_O_8_ (2.45 mM, concentration) in the dark for 16 hr and adjusting the absorbance at 734 nm to 0.70 with ethanol. Thereafter, 0.2 ml of appropriate dilution of the extract was added to 2.0 ml ABTS solution and the absorbance was measured at 734 nm after 15 min.

### 2.8 Hydroxyl radical scavenging ability

The hydroxyl radical scavenging activity of the extract was determined by the method of Halliwell, Gutteridge, and Aruoma (1987). Briefly, various concentrations of the extracts were mixed with 1 ml of reaction mixture (100 μM FeCl_3_, 104 μM EDTA, 1.5 mM H_2_O_2_, 2.5 mM deoxyribose and 100 μM ascorbic acid in 10 mM KH_2_PO_4_–KOH, pH7.4) and incubated for 1 hr at 37°C. Thereafter, 1 ml of 0.5% TBA in 0.025N NaOH and 1 ml of 2.8% TCA was added to the mixture and heated for 30 min at 80°C, before reading the absorbance at 532 nm.

### 2.9 Ferric reducing antioxidant property

The method described by Oyaizu (1986) was used. In total, 2.5 ml aliquot was mixed with 2.5 ml 200 mM sodium phosphate buffer (pH 6.6) and 2.5 ml 1% potassium ferricyanide. The mixture was incubated at 50°C for 20 min and then 2.5 ml 10% trichloroacetic acid was added. This mixture was centrifuged at 650 rpm for 10 min. Thereafter, 5 ml of the supernatant was mixed with an equal volume of water and 1 ml 0.1% ferric chloride. The absorbance was measured at 700 nm.

### 2.10 Fe^2+^ chelating assay

The method of Puntel, Nogueira, and Rocha (2005) was employed. Briefly, 150 μl of freshly prepared 500 μM FeSO_4_ was added to a reaction mixture containing 168 μl of 0.1 M Tris‐HCl (pH 7.4), 218 μl saline and the extract at different concentrations. The reaction mixture was incubated for 5 min, before addition of 13 μl of 0.25% 1.10‐Phenanthroline (w/v). The absorbance was measured at 510 nm in the spectrophotometer.

### 2.11 NO radical scavenging ability

The reaction mixture (3 ml) containing sodium nitroprusside (10 mM) in phosphate buffered saline (PBS) and the extract at different concentrations were incubated at 25°C for 150 min. At every 30 min interval, 0.5 ml of the incubated sample was removed and 0.5 ml of Griess reagent (1% sulfanilamide, 0.1% naphthylethylene diamine dihydrochloride in 2% H_3_PO_4_) was added. The absorbance of the chromophore formed was measured at 546 nm (Mondal, Chakraborty, Gupta, & Muzumdar, 2006).

### 2.12 α‐Amylase inhibitory activity

This was determined according to the method of Worthington (1993). Phenolic extract dilutions of 0–150 μl and 500 μl of 0.02 M sodium phosphate buffer (at pH of 6.9 with 6 mM NaCl) containing porcine pancreatic amylase (EC 3.2.1.1) (0.5 mg/ml) were incubated at 25°C for 10 min. Thereafter 500 μl of 1% starch solution in 0.02 M sodium phosphate buffer (pH 6.9 with 6 mM NaCl) was added to each tube. The reaction mixture was incubated at 25°C for 10 min and stopped with the addition of dinitrosalicylic acid (DNSA). The mixture was then incubated in a boiling water bath for 5 min and cooled at room temperature (24 ± 1°C). Finally, 10 ml of distilled water was added to the reaction mixture, and absorbance was read at 540 nm in the JENWAY UV‐Visible spectrophotometer.

### 2.13 α‐Glucosidase inhibitory activity

This was carried out according to the method of Apostolidis, Kwon, and Setty (2007). Phenolic extract dilutions of 1–150 and 100 μl of *α*‐glucosidase (EC 3.2.1.20) solution (1.0 U/ml) in 0.1 M phosphate buffer (pH 6.9) were incubated at 25***°***C for 10 min. Thereafter, 50 μl of 5 mM para‐nitrophenyl‐*α*‐D‐glucopyranoside solution (pNPG) in 0.1 M phosphate buffer (pH 6.9) was added. Finally, the reaction mixture was incubated at 25***°***C for 5 min, before reading the absorbance at 405 nm in the JENWAY UV‐Visible spectrophotometer.

### 2.14 Acetylcholinesterase and butyrylcholinesterase inhibitory activities

Inhibition of AChE was determined by a modified colorimetric method of Ellman, Courtney, Andres, and Featherstone (1961). The AChE activity was determined in a reaction mixture containing 200 μl of a solution of AChE (0.415 U/ml in 0.1 M phosphate buffer, pH 8.0), 100 μl of a solution of 5,5‐dithiobis(2‐nitrobenzoic) acid (3.3 mM DTNB in 0.1 M phosphate buffered solution, pH 7.0, containing 6 mM NaHCO_3_), 30 μl the plant extract and 500 μl of phosphate buffer, pH 8.0. After incubation for 20 min at 25°C, 100 μl of 0.05 mM acetylthiocholine iodide solution was added as substrate, and AChE activity was determined as changes in absorbance at 412 nm for 3 min at 25°C using a spectrophotometer. 100 μl of butyrylthiocholine iodide was used as substrate to assay butyrylcholinesterase activity, while all other reagents and conditions were the same. The AChE and BChE inhibitory activities were expressed as percentage inhibition.

### 2.15 Data analysis

All data were expressed as the mean ± standard error of means (SEM). Differences were assessed by a one‐way analysis of variance (ANOVA) followed by the *post hoc* LSD test for analysis of biochemical data using SPSS version 10.0 (SPSS Inc., Chicago, IL, USA) statistical software. *p* < 0.05 was considered statistically significant, while differences between groups of HPLC were assessed by an analysis of variance model and Tukey's test. The level of significance for the analyses was set to *p* < 0.05. These analyses were performed using the free software R version 3.1.1 (Zar, 2009).

## 3 RESULTS

The quantification of total phenol and total flavonoid of *S. biafrae* leaf are presented in Table 1. The total phenol content was significantly (*p* < 0.05) higher than total flavonoid.

**Table 1:** Total phenol and total flavonoids of phenolic extract of *Senecio biafrae* leaf

Total phenol (mg gallic acid equivalent/100 g dry weight extract)	Total flavonoid (mg quercetin equivalent/100 g dry weight extract)
201.26 ± 1.47^b^	41.19 ± 2.16^a^

*Note*

Values represent mean ± standard error of means (SEM) of triplicate readings.

Values with difference superscript across the row are significantly different (*p* > 0.05).

**Figure 1:**
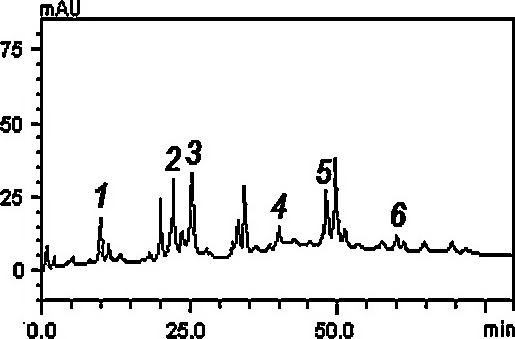
High‐performance liquid chromatography phenolics and flavonoids profile of *Senecio biafrae* extract. Gallic acid (peak 1), chlorogenic (peak 2), caffeic acid (peak 3), rutin (peak 4), quercetin (peak 5), and kaempferol (peak 6)

The HPLC fingerprinting of the phenolic extract (Figure 1 and Table 2) revealed the presence of gallic acid (retention time‐*t*_R_ = 9.85 min, peak 1), chlorogenic acid (*t*_R_ = 22.07 min, peak 2), caffeic acid (*t*_R_ = 25.01 min, peak 3), rutin (*t*_R_ = 40.13 min, peak 4), quercetin (*t*_R_ = 48.59 min, peak 5) and kaempferol (*t*_R_ = 60.15 min, peak 6). The HPLC analysis also revealed that the flavonoids (like quercetin, rutin and kaempferol) and phenolic acids (such as chlorogenic, gallic and caffeic acids) were the components of the extract.

**Table 2:** Phenolic compositions of *Senecio biafrae* extract by HPLC‐DAD

Compounds	Quantity (mg/g)
Gallic acid	1.46 ± 0.01^c^
Chlorogenic acid	2.73 ± 0.03^e^
Caffeic acid	3.11 ± 0.10^f^
Rutin	0.65 ± 0.11^b^
Quercetin	1.85 ± 0.02^d^
Kaempferol	0.36 ± 0.04^a^

*Note*

Results are expressed as mean ± SEM of three determinations.

Different letters in each column represent significant differences (*p* < 0.05).

**Figure 2:**
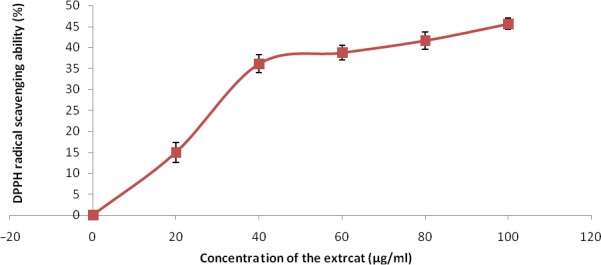
DPPH free radical scavenging ability of phenolic extract of Senecio biafrae leaf.Values are represented as mean ± SEM of triplicate experiments

**Figure 3:**
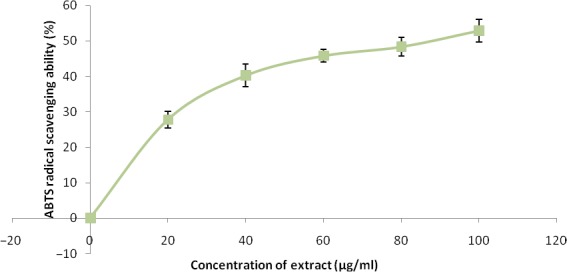
ABTS free radical scavenging ability of phenolic extract of Senecio biafrae leaf. Values are represented as mean ± SEM of triplicate experiments

**Figure 4:**
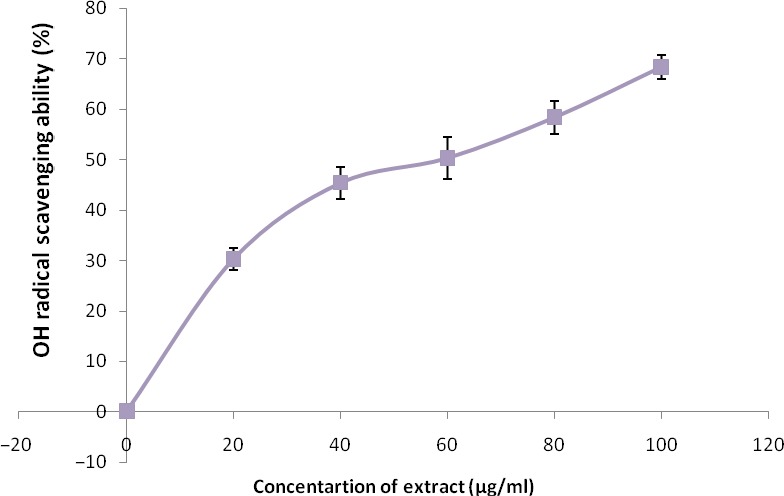
OH radical scavenging ability of phenolic extract of Senecio biafrae leaf. Values are represented as mean ± SEM of triplicate experiments

**Figure 5:**
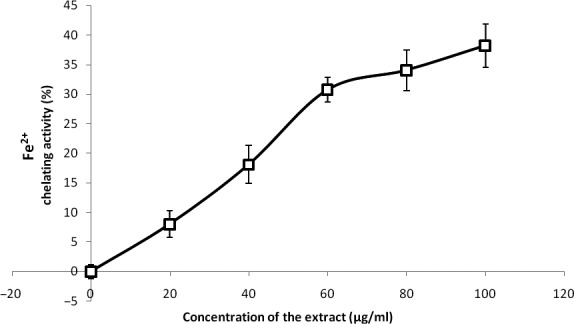
Fe^2+^ chelating ability of phenolic extract of Senecio biafrae leaf. Values are represented as mean ± SEM of triplicate experiments

**Figure 6:**
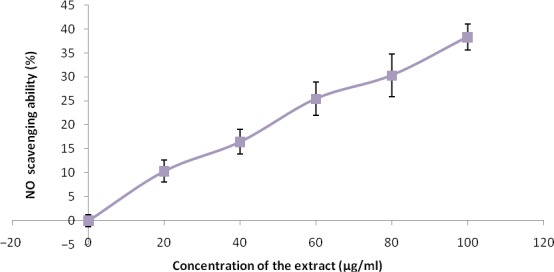
NO scavenging ability of phenolic extract of Senecio biafrae leaf. Values are represented as mean ± SEM of triplicate experiments

The free radical scavenging abilities are presented in Figures 2, 3, 4, 5, 6 and Table 3. The extract demonstrated inhibitory abilities against all free radicals in concentration‐dependent manner with IC_50_ (Table 4) ranging from 65.02 to 127.23 μg/ml.

**Table 3:** Ferric reducing antioxidant power (FRAP) (ascorbic acid equivalent mg/100 g) of *Senecio biafrae* extract

Extract	Inhibitory
Phenolic extract of *S. biafrae*	474.25 ± 2.23

*Note*

Values are represented as mean ± SEM of triplicate experiments.

**Table 4:** IC_50_ values for the in vitro antioxidant properties and enzymes inhibitory ability of phenolic extract of *Senecio biafrae* leaf

	IC_50_ values (μg/ml)
Fe^2+^ chelating ability	118.76
OH radical scavenging ability	65.02
ABTS radical scavenging ability	78.25
DPPH free radical scavenging ability	92.08
NO radical scavenging ability	127.23
α‐Amylase inhibition	126.90
α‐Glucosidase inhibition	139.66
Acetylcholinesterase inhibition	347.22
Butrylrycholinesterase inhibition	378.79

**Figure 7:**
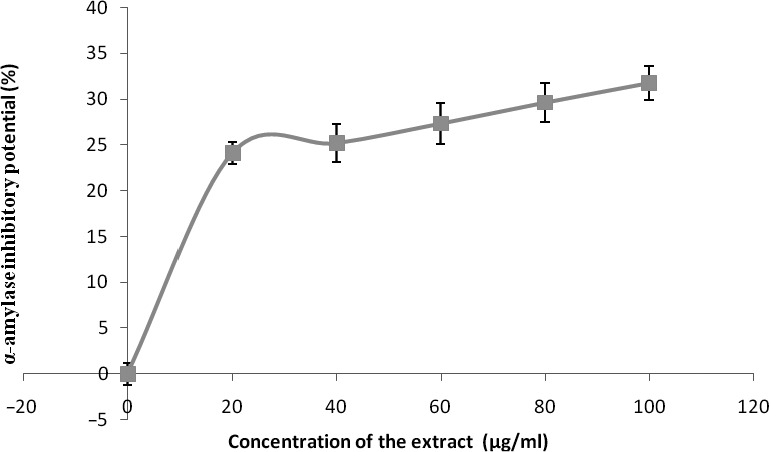
Inhibitory activities of phenolic extract of Senecio biafrae leaf against alpha‐amylase. Values are represented as mean ± SEM of triplicate experiments

**Figure 8:**
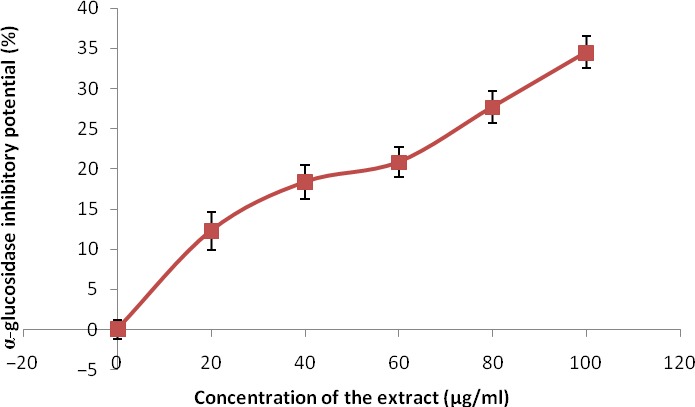
Inhibitory activities of phenolic extract of Senecio biafrae leaf against alpha‐glucosidase. Values are represented as mean ± SEM of triplicate experiments

**Figure 9:**
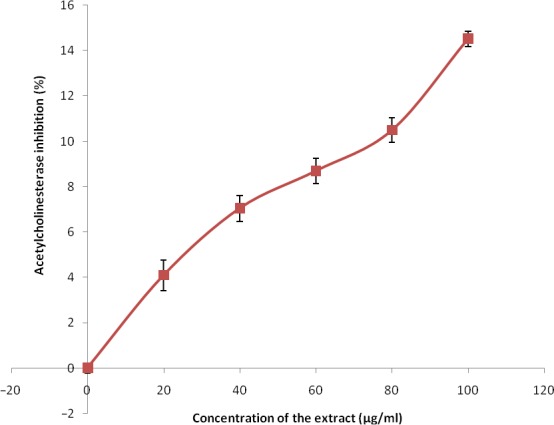
Acetylcholinesterase inhibition by phenolic extract of Senecio biafrae leaf. Values are represented as mean ± SEM of triplicate experiments

**Figure 10:**
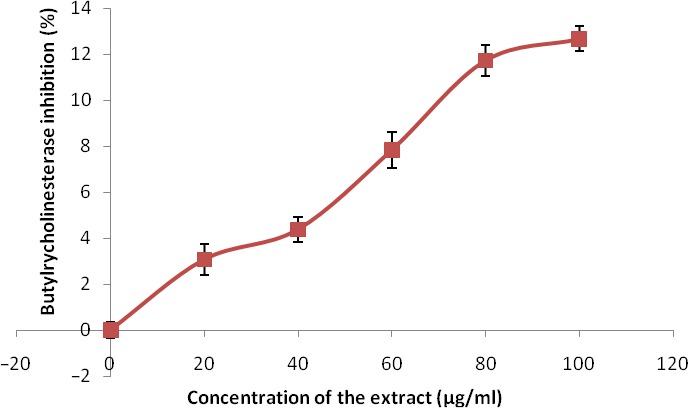
Butylrylcholinesterase inhibition by phenolic extract of Senecio biafrae leaf. Values are represented as mean ± SEM of triplicate experiments

The phenolic extract also demonstrated significant (*p* < 0.05) inhibitory activities against α‐amylase and α‐glucosidase (Figures 7 and 8) with IC_50_ (Table 4) between 126.90 and 139.66 μg/ml, respectively. Figures 9 and 10 show the inhibitory activities against acetylcholinesterase and butylrycholinestearse. The results showed that the extract was able to inhibit acetylcholinesterase and butylrycholinestearse in concentration‐dependent manner with IC_50_ of 347.22 and 378.79 μg/ml, respectively.

## 4 DISCUSSION

The antioxidative potential of phenolic compounds in protecting human body system from free radicals, has been reported by Saliu and Olabiyi (2017). The phenolics in the extract were capable of removing free radicals, chelate metallic catalysts, activate antioxidant enzymes, reduce alpha tocopherol radicals and inhibit oxidases (Amic, Davidovic, Beslo, & Trinajstic, 2003). They may also contribute to the quality of food by modifying taste, aroma, color and flavor (Memnune et al., 2009). According to Oboh (2006), the phenolic constituents of plants are antioxidants capable of preventing the production of free radicals and/or scavenging free radicals produced in the body. The presence of flavonoids and phenolics (gallic acid, chlorogenic, caffeic acid, rutin, quercetin and kaempferol) in the extract may also contribute to lowering cellular oxidative stress and inhibit α‐amylase, α‐glucosidase, acetylcholinesterase and butylrycholinesterase activities (Adefegha & Oboh, 2015).

Furthermore, Alam et al. (2014) documented that there is a link between the pathogenesis of type II diabetes mellitus and Alzheimer's disease with free radical. Plant bioactive compounds like phenolics, as observed in this study, may be useful for prevention and management of both type II diabetes mellitus and Alzheimer's disease, due to antioxidant nature of the extract.

Hydroxyl radicals are highly reactive and capable of damaging biomolecules (Adefegha & Oboh, 2015) via lipid peroxidation of biological membrane and damage to DNA. Hence the results indicate that the phenolics in the extract have scavenging properties. FRAP results in this study, demonstrate antioxidant activity of the extract. This property may enable the extract to mop up toxic metabolites released during pathological states and confer protection on the affected organs (Ajiboye et al., 2016).

Chelating iron might be useful in preventing generation of hydroxyl radicals. Oboh, Raddatz, and Henle (2008) reported that iron may serve as a metal catalyst in producing hydroxyl radicals from H_2_O_2_. The results of the present study showed that the extract chelated Fe^2+^, indicating that the generation of hydroxyl radicals in Fenton reaction can be attenuated and possible damage to biomolecules may be prevented.

Sivakumar, Mohandass, and Devika (2012) reported that nitric oxide is a free radical produced in mammalian cells, involved in the regulation of various physiological processes. Whereas, excess production of NO is associated with several diseases (e.g., diabetes mellitus, Alzheimer's disease, cancer, etc.). The results showed nitrite production was reduced by the phenolic extract (gallic acid, chlorogenic, caffeic acid, rutin, quercetin, and kaempferol).

Abirami, Nagarani, and Siddhuraju (2014) reported that diabetes mellitus is characterized by high concentration of blood sugar which can cause serious complications in the kidneys, liver, eyes, brain and cardiovascular system. The treatment of diabetes mellitus (especially in type II) focus mainly on reducing fluctuations in blood sugar and its complications (Sharifi‐Rad et al., 2016). Therefore, inhibition of enzymes involved in the metabolism of carbohydrates, mainly α‐amylase and α‐glucosidase are key therapeutic approaches for decreasing hyperglycemia (Sharifi‐Rad et al., 2016). In this study, the phenolic extract strongly inhibited α‐amylase and α‐glucosidase. This may suggest that inhibition activities against α‐amylase and α‐glucosidase could be part of the possible mechanisms of the phenolic extract in dietary management of diabetes, by retarding the starch and oligosaccharides hydrolysis in the gastrointestinal tract. This would in turn cause a decrease in the absorption of glucose and consequently inhibit the increase in postprandial blood glucose (Krentz & Bailey, 2005). These dual inhibitory potentials against the target enzymes might be due to the presence of phenolics and flavonoids (gallic acid, chlorogenic, caffeic acid, rutin, quercetin, and kaempferol) in the plant extract used.

Oxidative stress has been implicated in the pathogenesis and progression of Alzheimer's disease, with loss of cholinergic neurons in the brain (Saliu & Olabiyi, 2017). The results revealed that leaf extract inhibited both AChE and BChE activities. The inhibitions of AChE and BChE activities have been established as an effective strategy for treatment of Alzheimer's disease (Howes, Perry, & Houghton, 2003). Oboh, Agunloye, Akinyemi, Ademiluyi, and Adefegha (2012) reported that inhibition of AChE and BChE activity prevents it from breaking down acetylcholine and butyrylcholine in the brain to increase the concentrations of the neurotransmitters at the synaptic cleft. This in turn leads to increase communication between the nerve cells consequently improving or stabilizing symptoms of Alzheimer's disease. Orhan et al. (2007) reported that cholinesterase inhibitory effect of polyphenolic compounds is a function of number and position of their hydroxyl (OH) groups that forms hydrogen bonds with specific amino acids at the enzymes active sites. Therefore, inhibition of AChE and BChE by phenolic extract of *S. biafrae* leaf indicates neuroprotective ability of the extract which may be attributed to gallic acid, chlorogenic, caffeic acid, rutin, quercetin and kaempferol in the extract (Oboh et al., 2016).

## 5 CONCLUSION

The various concentrations of phenolic extract of *S. biafrae* leaf demonstrate that they not only possess antioxidant and free radical scavenging activities but also exhibit inhibitory potential against α‐amylase, α‐glucosidase, acetylcholinesterase, and butyrylcholinesterase. Thus, this extract may be promising for the therapeutic approach in the management of both type II diabetes mellitus and Alzheimer's disease due to its neutraceutical potentials.

## ETHICAL STATEMENT

The uses of either humans or animals were not applicable in this study.

## CONFLICT OF INTEREST

We declare that we have no conflict of interest.
